# Higher nonunion rates with locking plates compared to dynamic compression plates in forearm diaphyseal fractures: a multicenter study

**DOI:** 10.1186/s10195-025-00823-4

**Published:** 2025-02-21

**Authors:** Tzu-Hao Tseng, Chih-Chien Hung, Hung-Kuan Yen, Ho-Min Chen, Chen-Yu Wang, Shi-Chien Tzeng, Shau-Huai Fu

**Affiliations:** 1 Department of Orthopedic Surgery, National Taiwan University Hospital, No.7 Chungsan South Road, Taipei, 10002 Taiwan; 2 Department of Orthopedics, National Taiwan University Hospital Yun-Lin Branch, No.579, Sec. 2, Yunlin Rd., Yunlin County 632, Douliu, Taiwan; 3 Health Data Research Center, National Taiwan University, Taipei, Taiwan; 4 National Center for Geriatrics and Welfare Research, National Health Research Institutes, Zhunan, Taiwan

**Keywords:** Forearm diaphyseal fracture, Locking plate, Dynamic compression plate, Nonunion, Fractures of the radius of the forearm, Fractures of the ulna of the forearm

## Abstract

**Background:**

Dynamic compression plate (DCP) osteosynthesis is the gold standard for treating forearm diaphyseal fractures, providing stability and promoting healing. Locking plates (LPs) are increasingly used in modern fracture management but may increase the risk of nonunion if applied with excessive rigidity and without proper fracture site compression. The purpose of this study is to compare the nonunion rate between LPs and DCPs.

**Materials and methods:**

We conducted a retrospective study by reviewing the medical records and radiographs of 515 patients diagnosed with radial and/or ulnar shaft fractures at three trauma centers between 2014 and 2019. Inclusion criteria were patients treated with locking plates (LPs), locking compression plates (LCPs), or dynamic compression plates (DCPs) who had at least 9 months of outpatient follow-up and imaging assessments. Exclusion criteria included treatment with other methods, hospitalization for pathological fractures or implant removal, or incomplete surgical records. Data on patient demographics, injury details, and surgical outcomes were collected to compare nonunion rates, as well as early and late complications, between the LP and DCP groups.

**Results:**

A total of 368 patients were included in the analysis. Among them, 132 (35.9%) had isolated radial shaft fractures, 116 (31.5%) had isolated ulnar shaft fractures, and 120 (32.6%) had both-bone fractures. Of these, 124 patients received LP implants, 98 were treated with LCPs, and 146 were treated with DCPs. Early complications were comparable among the groups; however, the nonunion rate was significantly higher in the LP group (18.5% versus 11.2% versus 6.2%, *p* < 0.007). Logistic regression identified LP use [odds ratio (OR): 3.05, 95% confidence interval (CI) 1.24–7.53] as a significant predictor of nonunion. Notably, LPs lacking dynamic compression functionality were associated with markedly higher odds of nonunion in radial shaft fractures (OR: 26.94, 95% CI 3.52–206.15). These findings collectively indicate that LPs increase the nonunion rate in forearm fractures.

**Conclusions:**

Using LPs without compression functionality to treat forearm diaphyseal fractures increases the nonunion rate, particularly in radial shaft fractures. Therefore, we recommend using LCPs or DCPs for forearm diaphyseal fractures to ensure adequate compression at the fracture site during fixation, thereby promoting optimal bone healing rates.

*Level of evidence*: Level III: retrospective comparative therapeutic study.

**Supplementary Information:**

The online version contains supplementary material available at 10.1186/s10195-025-00823-4.

## Introduction

Forearm diaphyseal fractures are not uncommon orthopedic injuries, with an average incidence of 1.35 per 10,000 population reported in adults [1]. The forearm plays a crucial role in upper limb function, particularly in pronation and supination movements, making effective management of these fractures essential for restoring optimal limb functionality. The primary goal of treating forearm fractures is to restore length, rotational alignment, and the anatomical curvature of the radius [2]. Plate osteosynthesis with a dynamic compression plate (DCP) is generally recognized as the gold standard [2–4]. The DCP provides absolute stability and promotes primary bone healing through direct cortical apposition and compression at the fracture site. Although the union rate with DCP treatment exceeds 90% [3, 5–8], subsequent studies have explored the use of alternative implants to further improve treatment outcomes [9–14].

Locking plates (LPs) have become essential tools in modern fracture treatment [15, 16], demonstrating effectiveness in managing complex fractures, including osteoporotic, periprosthetic, and metaphyseal fractures [17]. Unlike DCPs, LPs provide greater stability by locking the screws into the plate, which generates greater resistance to shearing forces and creates a monoblock effect of the screw-plate construct [18–20]. Some LPs are designed to retain the compression function of the original DCP and are referred to as locking compression plates (LCPs) [11, 13, 14]. These plates allow for moderate compression at the fracture site before being secured with locking screws. In contrast, other LPs lack the compression function entirely. The success of DCPs in treating forearm fractures is based on achieving both fracture site compression and rigid fixation, which facilitates primary bone healing. Theoretically, simply increasing construct stability without providing fracture compression might hinder bone healing. This raises potential concerns regarding the use of noncompression LPs in the treatment of forearm fractures [16].

LPs are also increasingly being used in the treatment of forearm diaphyseal fractures [11, 12, 14, 15, 21], with the LCP being one of the more frequently utilized options [11, 12, 14]. Although several studies with limited patient numbers have shown that LCP treatment for forearm fractures can yield results comparable to those of conventional plates [11–14], these studies may be underpowered. Moreover, locking plates that do not provide fracture site compression have not been thoroughly investigated. Therefore, whether the use of LPs in treating forearm diaphyseal fractures can achieve fracture union rates comparable to those of conventional plates remains a topic that warrants further exploration.

In this study, our objective is to compare the nonunion rates between the use of LPs and DCPs in the treatment of forearm diaphyseal fractures. We hypothesize that the treatment outcomes with LPs will not be superior to those achieved with conventional DCPs, and that LPs unable to achieve fracture site compression will have the highest risk of nonunion.

## Materials and methods

After obtaining approval from the Research Ethics Committee (REC) of the National Health Research Institutes (REC approval number:NTUH-REC no. 202012043RINB ), we conducted a retrospective multicenter comparative study to evaluate nonunion in patients with radial and/or ulnar shaft fractures treated with LPs, LCPs, or DCPs. This study adhered to the Strengthening the Reporting of Observational Studies in Epidemiology (STROBE) guidelines for observational studies.

**Fig. 1:**
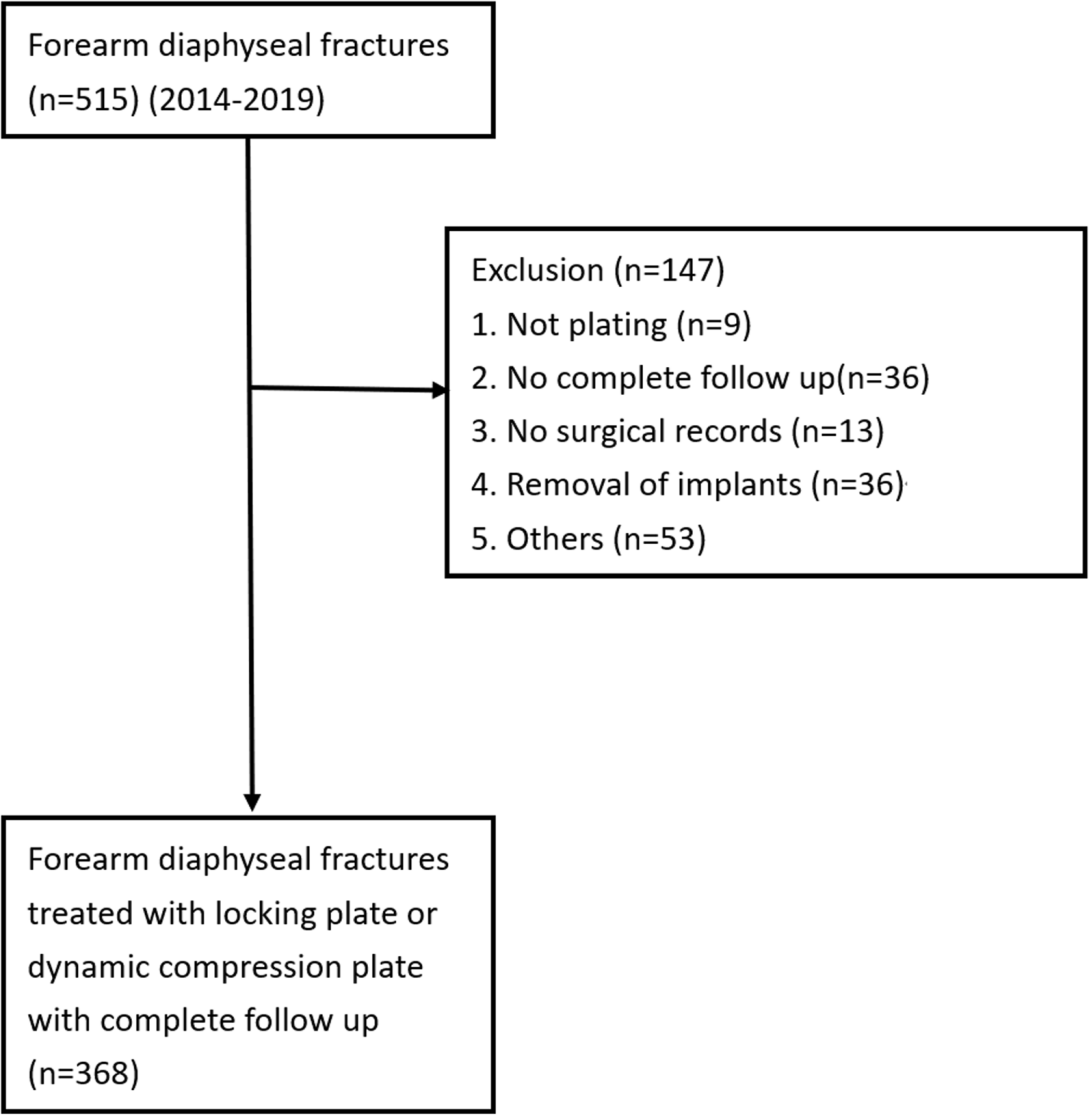
Flowchart of this study

As the objective of this study was to analyze adult forearm diaphyseal fractures, we reviewed 515 electronic medical records of patients aged 20 years or older who were admitted to three level I trauma centers (hospital 1: National Taiwan University Hospital (NTUH), hospital 2: NTUH Hsin-Chu Branch, and hospital 3: NTUH Yun-Lin Branch) with diagnoses of radial shaft fracture, ulnar shaft fracture, or both-bone forearm fractures between 2014 and 2019 (Fig. 1). Only cases treated with DCPs or LPs, with imaging follow-up and outpatient follow-up of at least 9 months, were included, while those treated with other methods, such as nails, wires, or external skeletal fixation, were excluded. Additional exclusion criteria included admissions for the treatment of pathological fractures, removal of implants, lack of surgical records, and other unspecified reasons. Patients who did not have at least 9 months of follow-up were also excluded. The 9-month period was chosen because, although there is no universally accepted time frame for defining nonunion in adults, it is a widely recognized threshold in many studies for determining nonunion [22, 23].

We collected baseline characteristics including age, gender, smoking history, and the hospital of admission for each patient. Comprehensive information related to the injury mechanism, length of stay, fracture type and classification, dislocation, whether the fracture was open or closed, implant type, the use of artificial bone substitute, the postoperative use of nonsteroidal antiinflammatory drugs (NSAIDs) or antiosteoporotic medications (AOM), and follow-up duration was also gathered. Two orthopedic surgeons (S.H.F. and C.C.H.) independently reviewed the radiographs of each patient. The Association of the Study of Internal Fixation/Orthopedic Trauma Association (AO/OTA) classification system was used to determine the initial fracture severity. Postoperative radiographs were reviewed to assess the presence of nonunion, which was defined as the primary outcome. Nonunion was defined as the presence of a fracture gap or the absence of progressive callus formation after 9 months postsurgery [24]. A third orthopedic surgeon (T.H.T.) was consulted in cases where the initial assessments were incongruent.

Secondary outcomes included early complications such as superficial surgical site infection (SSSI), deep surgical site infection (DSSI), and fixation failure or loss of reduction within 3 months. Late complications were also recorded, including chronic surgical site infection (CSSI), regional pain syndrome, joint contracture, malunion, and fixation failure after 3 months. In addition, removal of implants (ROI) and refracture after ROI were documented.

### Statistical analysis

We employed the chi-squared test for analyzing categorical variables and the analysis of variance (ANOVA) test for continuous variables. A logistic regression model was used to assess the risk factors associated with nonunion. Statistical analyses were conducted using SAS software, version 9.4 (SAS Institute, Cary, NC, USA), with two-sided tests applied at a significance level of *α *= 0.05. Sample size calculation and post hoc power analysis were performed for nonunion rates using G*power software, version 3.1.9.7 (Heinrich-Heine-Universitat Dusseldorf, Dusseldorf, Germany).

## Results

A total of 368 patients were included in the analysis (Fig. 1), with baseline characteristics detailed in Table 1. Patients were categorized into three groups on the basis of the implant used: LP (locking plate without compression function), LCP, and DCP. Among them, 124 patients were classified into the LP group, 98 into the LCP group, and 146 into the DCP group.

**Table 1:** Baseline characteristics

		Total (*N* = 368)		LP (*N* = 124)		LCP (*N* = 98)		DCP (*N* = 146)		*P*-value*
		*n*	(%)	*n*	(%)	*n*	(%)	*n*	(%)	
Age, years	Mean (SD)	43.7	(18.6)	44.4	(18.4)	46.5	(18.1)	41.2	(18.9)	0.083
	Median (Q1–Q3)	39	(27–60)	43	(27–62)	44	(32–60)	38	(23–58)	
	Range (years)	20–87		20–86		20–81		20–87		
Sex (male)		230	(62.5)	81	(65.3)	45	(45.9)	104	(71.2)	< 0.001
Hospital	Hospital 1	142	(38.6)	52	(41.9)	44	(44.9)	46	(31.5)	< 0.001
	Hospital 2	114	(31.0)	15	(12.1)	44	(44.9)	55	(37.7)	
	Hospital 3	112	(30.4)	57	(46.0)	10	(10.2)	45	(30.8)	
High energy		283	(76.9)	94	(75.8)	74	(75.5)	115	(78.8)	0.788
Mechanism	Fall from standing height	73	(19.8)	27	(21.8)	21	(21.4)	25	(17.1)	0.596
	Fall from height > 1 m	24	(6.5)	8	(6.5)	4	(4.1)	12	(8.2)	
	Traffic accident	206	(56.0)	69	(55.6)	61	(62.2)	76	(52.1)	
	Machine injury	43	(11.7)	15	(12.1)	7	(7.1)	21	(14.4)	
	Sports injury	13	(3.5)	3	(2.4)	3	(3.1)	7	(4.8)	
	Others	9	(2.4)	2	(1.6)	2	(2.0)	5	(3.4)	
Smoking		73	(19.8)	21	(16.9)	15	(15.3)	37	(25.3)	0.095
Length of hospital stay, days	Mean (SD)	5.6	(6.0)	5.9	(6.6)	4.6	(3.4)	5.9	(6.6)	0.692
	Median (Q1–Q3)	4	(3–6)	4	(3–6)	4	(3–5)	4	(3–6)	
Fracture type	Both-bone	120	(32.6)	47	(37.9)	39	(39.8)	34	(23.3)	0.037
	Isolated radial shaft	132	(35.9)	40	(32.3)	34	(34.7)	58	(39.7)	
	Isolated ulna shaft	116	(31.5)	37	(29.8)	25	(25.5)	54	(37.0)	
Dislocation	(Yes)	76	(20.7)	17	(13.7)	25	(25.5)	34	(23.3)	0.059
	- Galeazzi	58	(15.8)	12	(9.7)	18	(18.4)	28	(19.2)	–
	- m Monteggia	18	(4.9)	5	(4.0)	7	(7.1)	6	(4.1)	
AO classification	22–1	125	(34.0)	44	(35.5)	27	(27.6)	54	(37.0)	0.400
	22–2	144	(39.1)	44	(35.5)	41	(41.8)	59	(40.4)	
	22–3	99	(26.9)	36	(29.0)	30	(30.6)	33	(22.6)	
Open fracture	(Yes)	33	(9.0)	10	(8.1)	14	(14.3)	9	(6.2)	0.085
	- Gustilo I	23	(6.3)	6	(4.8)	10	(10.2)	7	(4.8)	
	- Gustilo II	7	(1.9)	2	(1.6)	4	(4.1)	1	(0.7)	
	- Gustilo III	3	(0.8)	2	(1.6)	0	(0.0)	1	(0.7)	
Artificial bone substitute		36	(9.8)	7	(5.6)	12	(12.2)	17	(11.6)	0.161
NSAID		306	(83.2)	103	(83.1)	82	(83.7)	121	(82.9)	0.986
AOM		7	(1.9)	1	(0.8)	3	(3.1)	3	(2.1)	0.467
Follow-up time, months	Mean (SD)	15.5	(13.0)	14.9	(12.3)	16.3	(12.0)	15.6	(14.3)	
	Range (months)	9–35		9–30		9–32		9–35		

Among the 368 patients, 120 had both-bone fractures, 132 had isolated radial shaft fractures, and 116 had isolated ulnar shaft fractures. Of the patients with both-bone fractures, 20 patients underwent the mixed approach. Among these, six patients had the radius fixed with an LCP and the ulna with a DCP, while one patient had the radius fixed with a DCP and the ulna with an LCP. On the basis of our hypothesis that the use of locking plates may influence the nonunion rate, these seven patients were categorized into the LCP group. In addition, 9 patients had the radius fixed with an LP and the ulna with a DCP, while 4 patients had the radius fixed with a DCP and the ulna with an LP; for the same reason, these 13 patients were categorized into the LP group. An individual bone-specific analysis was also performed. For a total of 252 radial shaft fractures, 83 cases were fixed with LPs, 72 with LCPs, and 97 with DCPs. For a total of 236 ulnar shaft fractures, 75 cases were fixed with LPs, 58 with LCPs, and 103 with DCPs.

The ages across the groups were comparable (*P* = 0.083), with an average age in the 40s. The proportion of male patients was significantly lower in the LCP group (*P* < 0.001). Moreover, fewer patients at hospital 2 (for hospital names, see title page) received LP implants, and fewer patients at hospital 3 received LCP implants compared with those at the other two hospitals (*P* < 0.001). Forearm two-bone fractures, radial shaft fractures, and ulnar shaft fractures each accounted for about one third of the cases in each. However, forearm two-bone fractures were less prevalent in the DCP group (23.3%, *P* = 0.037). No significant differences were observed between the three groups in terms of smoking history, injury mechanism, length of hospital stay, fracture classification, open fractures, artificial bone substitute use, or NSAID use. The artificial bone substitute was composed of hydroxyapatite. The implant used in the DCP group was the 3.5 mm Dynamic Compression Plate (DePuy Synthes, MA, USA). In the LCP group, the Small Fragment Locking Compression Plate (DePuy Synthes, MA, USA) was used. In the LP group, four types of LPs were utilized. Three were 3.5 mm straight locking plates, manufactured by different brands, including A-plus (Taiwan), Civic (Taiwan), and the Zimmer Biomet Small Fragment Universal Locking System (IN, USA). The fourth was the Acumed Anatomic Midshaft Forearm Plate (OR, USA). Regarding materials, the DCPs were made of stainless steel, whereas both the LCPs and LPs were made of titanium.

Complications are detailed in Table 2. No iatrogenic vascular injuries or vessel damage were reported during surgery (data not shown). A small percentage of patients (3.3%) experienced early complications (Table 2), including SSSI (0.5%), DSSI (1.1%), and fixation failure/loss of reduction (1.6%) within the first 3 months. The early complication rate did not differ significantly between the groups. However, late complications were significantly different among the three groups (25.0% versus 15.3% versus 11.0%, *P* = 0.008). When these late complications were further analyzed, there were no significant differences between the groups in terms of CSSI, regional pain syndrome, joint contracture, malunion, or fixation failure. The nonunion rates, however, were significantly different among the three groups (18.5% versus 11.2% versus 6.2%, *P* = 0.007). A pairwise chi-squared test with Bonferroni correction revealed that the difference was only significant between the LP and DCP groups (18.5% versus 6.2%, *P* = 0.003). Regarding ROI and refracture after ROI, no significant differences were observed between the groups. The results remained consistent even when the analysis was conducted on the basis of individual bones rather than individual patients (Supplementary Table 1). The LP group still had a significantly higher rate of nonunion (16.1% versus 9.6% versus 5.0%* P* = 0.002).

**Table 2:** Frequency of outcome variables for patient data

		Total (*N* = 368)		LP (*N* = 124)		LCP (*N* = 98)		DCP (*N* = 146)		*P*-value
		*n*	(%)	*n*	(%)			*n*	(%)	
Early complication		12	(3.3)	4	(3.2)	3	(3.1)	5	(3.4)	0.987
- SSSI		2	(0.5)	0		1	(1.0)	1	(0.7)	–
- DSSI		4	(1.1)	4	(3.2)	0		0		
- Fixation failure/loss of reduction		6	(1.6)	0		2	(2.1)	4	(2.7)	
Late complication		62	(16.8)	31	(25.0)	15	(15.3)	16	(11.0)	0.008
- Chronic surgical site infection		3	(0.8)	3	(2.4)	0		0		0.051
- Regional pain syndrome		0		0		0		0		–
- Joint contracture		1	(0.3)	0		1	(1.0)	0		0.251
- Malunion		0		0		0		0		–
- Fixation failure		15	(4.1)	5	(4.0)	3	(3.1)	7	(4.8)	0.798
- Nonunion		43	(11.7)	23	(18.5)	11	(11.2)	9	(6.2)	0.007
Removal of implants		111	(30.2)	31	(25.0)	34	(34.7)	46	(31.5)	0.266
Refracture after ROI		11	(3.0)	3	(2.4)	2	(2.0)	6	(4.1)	0.584

After conducting a logistic regression analysis with stepwise variable selection, only the use of LPs was found to be significantly associated with nonunion (Table 3). The LP group had a significantly higher odds ratio (OR) for nonunion (3.05, 95% CI 1.24–7.53). The results remained consistent even when the analysis was conducted on the basis of individual bones rather than individual patients (Supplementary Table 2). The OR for nonunion of the LP group was 3.97 (95% CI 1.54–10.21) at the bone level.

**Table 3:** Odds ratio of nonunion

	Crude OR			Adjusted OR		
	OR	(95% CI)	*P*-value	OR	(95% CI)	*P*-value
Age						
≤ 60	Ref.		0.1330	Ref.		0.188
> 60	1.70	(0.85–3.38)		1.70	(0.77–3.72)	
Sex (male versus female)	1.14	(0.58–2.21)	0.7062	1.43	(0.64–3.19)	0.386
Hospital						
Hospital 1	Ref.		0.4361	Ref.		0.645
Hospital 2	0.71	(0.31–1.61)		0.67	(0.27–1.67)	
Hospital 3	1.23	(0.59–2.55)		1.04	(0.44–2.50)	
High energy versus low energy	0.99	(0.47–2.10)	0.9791	1.74	(0.53–5.70)	0.358
Mechanism						0.366
Fall from height	Ref.		0.6192	Ref.		
Traffic accident	0.71	(0.35–1.45)		0.47	(0.16–1.40)	
Others	0.72	(0.27–1.88)		0.48	(0.14–1.69)	
Smoking	1.26	(0.59–2.69)	0.5503	1.59	(0.67–3.79)	0.298
Stay						0.826
≤ 4 days	Ref.		0.6391	Ref.		
> 4 days	1.17	(0.61–2.23)		0.92	(0.45–1.89)	
Fracture type						
Ulna shaft	Ref.		0.0488	Ref.		0.050
Radius shaft	0.71	(0.30–1.71)		1.07	(0.14–8.28)	
Radius and ulna shaft	1.84	(0.86–3.93)		4.76	(0.87–26.23)	
Dislocation (yes versus no)	0.72	(0.31–1.69)	0.4530	1.21	(0.46–3.20)	0.704
AO classification						
22–1	Ref.		0.3469	Ref.		0.368
22–2	0.73	(0.33–1.60)		0.70	(0.11–4.31)	
22–3	1.31	(0.61–2.83)		0.35	(0.07–1.77)	
Open fracture (yes versus no)	1.40	(0.51–3.83)	0.5169	0.79	(0.24–2.66)	0.705
Implant						
DCP	Ref.		0.0096	Ref.		0.050
LCP	1.93	(0.77–4.83)		2.02	(0.74–5.51)	
LP	3.47	(1.54–7.81)		3.05	(1.24–7.53)	
Artificial bone substitute	1.60	(0.62–4.09)	0.3310	2.07	(0.69–6.21)	0.193
NSAID	0.87	(0.38–1.98)	0.7434	0.88	(0.36–2.17)	0.777

Factors, including age, sex, hospital, high-energy injury, injury mechanism, smoking, length of hospital stay, dislocation, fracture classification, open fractures, artificial bone substitute use, and NSAID use, did not show a significant association with our primary outcome. We also tested for interaction between forearm two-bone fractures and implant type, but no significant interaction was found between these two factors. After stratifying by fracture type (Table 4), LPs used for radial shaft fractures showed significantly higher OR for nonunion (26.94, 95% CI 3.52–206.15).

**Table 4:** Odds ratio of implant type, stratified by fracture type

	Ulna shaft			Radius shaft		
	OR	(95% CI)	*P*-value	OR	(95% CI)	*P*-value
DCP or LP						
DCP	Ref.		0.7058	Ref.		0.0008
LCP	1.54	(0.54–4.39)		7.31	(0.83–64.00)	
LP	1.09	(0.39–3.06)		26.94	(3.52–206.15)	

Using G*Power software for sample size calculation, with the alpha error probability set at 0.05 and power at 80%, the required sample size for each group, based on the nonunion rates of LP and DCP, was at least 88 participants. In our study, the sample sizes for the groups exceeded this threshold. In addition, a post hoc power analysis of the two-sample proportion *Z*-test for comparing the nonunion rates between the LP and DCP groups demonstrated a power of 92.5%, indicating that the study’s primary endpoint—the impact of LPs on the nonunion rate—had high statistical power.

## Discussion

The most important finding of this study is that treating forearm diaphyseal fractures with LPs does not yield better outcomes than conventional DCPs and is associated with a higher risk of nonunion, particularly when using LPs without dynamic compression functionality. Furthermore, the effect of LPs on increasing the nonunion rate is most pronounced in radial shaft fractures. Therefore, we recommend using LCPs or DCPs for treating such fractures to ensure better healing outcomes.

Dynamic compression plating has been considered the gold standard for treating forearm diaphyseal fractures, significantly improving treatment outcomes, with union rates exceeding 90% [3, 5–8]. The success of this method relies on meeting three essential conditions: anatomic reduction, appropriate fracture site compression, and rigid fixation, all of which facilitate fracture union through primary bone healing. LPs, with their angular-stable screw-plate interface and the enhanced pullout strength of locking head screws, provide greater stability [19, 20]. The design of LCPs allows surgeons to combine the benefits of locked plating and compression plating into a single implant [15]. Theoretically, if surgeons can achieve the same three conditions, LCPs should also successfully treat forearm diaphyseal fractures. However, there is a risk that the surgeon could unintentionally undermine the theoretical benefits, resulting in conditions at the fracture site that promote excessive gap strains and ultimately lead to nonunion [25]. This risk is especially pronounced when using LPs without dynamic compression functionality, as the likelihood of nonunion is significantly higher owing to insufficient fracture site compression [17]. Our study results clearly demonstrate that LPs used to treat radial shaft fractures have the highest OR for nonunion.

In literature, the aseptic nonunion rate for treating forearm diaphyseal fractures with DCPs ranges from 2 to 10% [2, 5–8, 26, 27]. In our study, the nonunion rate in the DCP group was 6.2%, which falls within this reasonable range, indicating that the surgical techniques used by the surgeons in our study met the expected standards. Regarding the comparison between LCP and DCP, several smaller studies have previously indicated that there is no significant difference in union rates between the two [11–14]. For example, Azboy et al. studied 22 patients with LCPs and 20 with DCPs, finding that after 21 months of follow-up, all fractures had healed with no difference in functional scores [14]. Similarly, Henle et al.’s study of 53 patients reported the same results, showing no difference in treatment outcomes between the two methods [12]. As mentioned earlier, these findings are reasonable because the LCP combines the advantages of both LPs and DCPs. If proper surgical techniques are applied, comparable outcomes should be expected. Compared with these previous studies, our strength lies in the larger patient cohort; to our knowledge, this is the largest forearm fracture cohort to date. Another strength of this study is that it included a comprehensive analysis of numerous factors that could influence bone healing, making it more representative than previous studies. While our study did show that LPs are associated with a higher rate of nonunion, this does not contradict previous literature. The significant increase in the OR for nonunion was observed only with nondynamic compression LPs. We believe that this is owing to the use of these LPs, which can create excessive rigidity at the fracture site when a gap is present, preventing both primary and secondary bone healing [17].

In this study, implant selection varied significantly among hospitals: fewer patients at hospital 2 received LP implants, while fewer at hospital 3 received LCP implants. Since existing literature does not definitively establish which type of plate is better suited for forearm diaphyseal fractures, no specific implant type was excluded during preoperative planning. The choice of implant was primarily influenced by two factors: (1) the surgeon’s preference and (2) the patient’s financial considerations. Both LP and LCP implants are relatively expensive in our country and are not covered by the national health insurance system. In addition, pricing varies among different manufacturers in our region. Consequently, implant selection was not randomly distributed but depended not only on the surgeon’s preference but also on the patient’s financial situation and their perceived value of the implant in relation to its cost. Moreover, as these three hospitals are located in different regions, variations in patients’ socioeconomic backgrounds likely contributed to the differences in implant selection. These factors may have introduced bias, highlighting the need for future randomized controlled trials to minimize this potential confounding effect.

This study has some limitations. First, as a retrospective study, we cannot be certain that all surgeons’ techniques met the expected standard. However, since the surgeons at these hospitals received the same orthopedic residency training and the nonunion rate was within a reasonable range, this limitation is likely minimal. Second, the retrospective nature of the study also led to the exclusion of 36 patients owing to incomplete follow-up, but the small proportion of these patients should have a negligible impact on the analysis and results. Third, this study did not set an upper age limit and included all adults aged 20 years or older. While the physiological healing of fractures theoretically varies across age groups, our analysis found no significant age differences among the three groups. Furthermore, multivariate analysis showed that age was not a significant factor influencing the nonunion rate in this study. A 9-month follow-up to confirm the nonunion rate is a widely accepted definition for adult fractures and does not impose any age restrictions [22, 23]. In addition, Galeazzi and Monteggia fractures have distinct mechanical characteristics in the wrist and elbow, differentiating them from forearm diaphyseal fractures. This distinction may influence the analysis results, and future prospective studies could exclude these fracture types for more accurate findings. Nonetheless, this study represents the largest retrospective cohort in current literature, underscoring the need for prospective studies to validate these results. Finally, the difference in materials is also a limitation of our study. Specifically, the DCP was made of stainless steel, whereas both the LCPs and LPs were made of titanium. This difference may have introduced bias into our results. To control this variable, a prospective study would be necessary.

## Conclusions

Using LPs without compression functionality to treat forearm diaphyseal fractures increases the nonunion rate, particularly in radial shaft fractures. Therefore, we recommend using LCPs or DCPs for forearm diaphyseal fractures to ensure adequate compression at the fracture site during fixation, thereby promoting optimal bone healing rates.

## Supplementary Information

Supplementary Material 1.

## Data Availability

The datasets used and/or analyzed during the current study are available from the corresponding author on reasonable request.

## Abbreviations

AOM: Antiosteoporotic medications
DCP: Dynamic compression plate
LCP: Locking compression plate
LP: Locking plate
NSAIDs: Nonsteroidal antiinflammatory drugs
OR: Odds ratio
