# Supplementary material for: Higher nonunion rates with locking plates compared to dynamic compression plates in forearm diaphyseal fractures: a multicenter study
Source: J Orthop Traumatol. 2025 Feb 21;26:10. doi: 10.1186/s10195-025-00823-4 (PMC11845636; doi:10.1186/s10195-025-00823-4)
Supplement: Supplementary file 1 — Supplementary Material 1. [file 10195_2025_823_MOESM1_ESM.docx]

**Supplementary Table 1. Frequency of outcome variables for individual bones**

|  | | *Total*  *(N=486)* | | *LP*  *(N=161)* | | *LCP*  *(N=125)* | | *DCP*  *(N=200)* | |  |
| --- | --- | --- | --- | --- | --- | --- | --- | --- | --- | --- |
|  |  | *n* | *(%)* | *n* | *(%)* |  |  | *n* | *(%)* | *p-value** |
| Early complication | | 18 | (3.7) | 7 | (4.3) | 3 | (2.4) | 8 | (4.0) | 0.660 |
| - SSSI | | 3 | (0.6) | 0 |  | 1 | (0.8) | 2 | (1.0) | - |
| - DSSI | | 8 | (1.6) | 7 | (4.3) | 0 |  | 1 | (0.5) |  |
| - fixation failure / loss of reduction | | 7 | (1.4) | 0 |  | 2 | (1.6) | 5 | (2.5) |  |
| Late complication | | 94 | (19.3) | 46 | (28.6) | 19 | (15.2) | 29 | (14.5) | 0.001 |
| - chronic surgical site infection | | 6 | (1.2) | 6 | (3.7) |  |  | 0 |  | 0.002 |
| - regional pain syndrome | | 0 |  | 0 |  | 0 |  | 0 |  | - |
| - joint contracture | | 2 | (0.4) | 0 |  | 1 | (0.8) | 1 | (0.5) | 0.559 |
| - malunion | | 0 |  | 0 |  | 0 |  | 0 |  | - |
| - fixation failure | | 17 | (3.5) | 6 | (3.7) | 4 | (3.2) | 7 | (3.5) | 0.972 |
| - non-union | | 48 | (9.9) | 26 | (16.1) | 12 | (9.6) | 10 | (5.0) | 0.002 |
| Removal of implants | | 152 | (31.3) | 44 | (27.3) | 46 | (36.8) | 62 | (31.0) | 0.229 |
| Refracture after ROI | | 13 | (2.7) | 12 | (7.5) | 6 | (4.8) | 6 | (3.0) | 0.932 |
| Revision | | 29 | (6.0) | 12 | (7.5) | 6 | (4.8) | 11 | (5.5) | 0.602 |

**Supplementary Table 2. Odds ratio of nonunion for individual bones**

|  | Crude OR | | |  | Adjusted OR | | |
| --- | --- | --- | --- | --- | --- | --- | --- |
|  | OR | (95% CI) | p-value |  | OR | (95% CI) | p-value |
| Age |  |  |  |  |  |  |  |
| <=60 | ref |  | 0.2378 |  | Ref |  | 0.287 |
| >60 | 1.52 | (0.76-3.06) |  |  | 1.55 | (0.70-3.46) |  |
| Sex (male vs female) | 1.14 | (0.59-2.21) | 0.7026 |  | 1.26 | (0.57-2.80) | 0.563 |
| Hospital |  |  |  |  |  |  |  |
| Hospital 1 | ref |  | 0.4807 |  | Ref |  | 0.822 |
| Hospital 2 | 0.66 | (0.29-1.49) |  |  | 0.75 | (0.31-1.83) |  |
| Hospital 3 | 1.08 | (0.52-2.25) |  |  | 0.93 | (0.38-2.25) |  |
| High energy vs low energy | 1.00 | (0.46-2.19) | 0.9821 |  | 2.19 | (0.63-7.54) | 0.215 |
| Mechanism |  |  |  |  |  |  |  |
| fall from height | ref |  | 0.6526 |  | Ref |  | 0.360 |
| traffic accident | 0.71 | (0.35-1.47) |  |  | 0.45 | (0.15-1.40) |  |
| Others | 0.76 | (0.29-2.01) |  |  | 0.48 | (0.13-1.73) |  |
| Smoking | 1.32 | (0.61-2.84 | 0.4796 |  | 1.48 | (0.60-3.62) | 0.391 |
| Length of Hospital Stay |  |  |  |  |  |  |  |
| <=4 days | ref |  | 0.8584 |  | Ref |  | 0.780 |
| >4 days | 0.94 | (0.49-1.80) |  |  | 0.90 | (0.44-1.87) |  |
| Fracture type |  |  |  |  |  |  |  |
| Ulna shaft | ref |  | 0.9308 |  | ref |  | 0.689 |
| Radial shaft | 1.03 | (0.55-1.91) |  |  | 1.19 | (0.51-2.76) |  |
| Dislocation (yes vs no) | 0.87 | (0.36-2.08) | 0.7473 |  | 1.14 | (0.42-3.12) | 0.799 |
| AO class |  |  |  |  |  |  |  |
| 22-1 | ref |  | 0.7829 |  | ref |  | 0.744 |
| 22-2 | 0.76 | (0.34-1.70) |  |  | 0.65 | (0.21-2.02) |  |
| 22-3 | 0.82 | (0.38-1.77) |  |  | 0.75 | (0.29-1.95) |  |
| Open fracture (yes vs no) | 0.89 | (0.34-2.36) | 0.8197 |  | 0.95 | (0.30-3.05) | 0.936 |
| Implant |  |  |  |  |  |  |  |
| DCP | ref |  | 0.0171 |  | ref |  | 0.022 |
| LCP | 2.04 | (0.78-5.36) |  |  | 2.41 | (0.84-6.90) |  |
| LP | 3.61 | (1.54-8.44) |  |  | 3.97 | (1.54-10.21) |  |
| Artificial bone substitute | 1.63 | (0.64-4.15) | 0.3085 |  | 2.23 | (0.72-6.98) | 0.166 |
| NSAID | 0.97 | (0.41-2.25) | 0.9333 |  | 0.92 | (0.36-2.35) | 0.856 |
